# Practical Medicine

**Published:** 1879-04

**Authors:** 


### PRACTICAL MEDICINE.

CHLORAL IN MALIGNANT CHOLERA.—(*Br. Med. Jour.*, Sept. 21, '78.)

Surgeon Major Hall of the Indian Army Service, reports at length a case of Asiatic cholera which had proceeded to a stage of extreme collapse, and which he successfully treated, as follows:

He used a solution of chloral, one in ten, and injected the equivalent of six grains, not simply under the skin, but deep into the substance of the muscles, changing the direction of the needle without withdrawing it from the skin. Improvement was manifest in a few minutes. In the course of eight hours thirty-two grains were thus injected, according to the urgency of the symptoms. Careful nursing and the exhibition of chloral *per os*, served to bring the case completely out of danger. Alcohol was scrupulously avoided. But little soreness followed the *deep* injections.

The writer argues that in this disease the arterial tension is much increased, and that as the cold stage comes on there is spasm of the muscular walls of the heart so strong that there is almost continuous systole. In deep, cholera collapse the heart sounds are inaudible, and pulse almost imperceptible. In such a condition of syncope as this, stimulants do absolute harm; their physiological antagonists are indicated, and hence the benefit rendered by the chloral in the above case.

